# Perception of Space by Multiple Intrinsic Frames of Reference

**DOI:** 10.1371/journal.pone.0010442

**Published:** 2010-05-03

**Authors:** Yanlong Sun, Hongbin Wang

**Affiliations:** School of Health Information Sciences, University of Texas Health Science Center at Houston, Houston, Texas, United States of America; L'université Pierre et Marie Curie, France

## Abstract

It has been documented that when memorizing a physical space, the person's mental representation of that space is biased with distortion and segmentation. Two experiments reported here suggest that distortion and segmentation arise due to a hierarchical organization of the spatial representation. The spatial relations associated with salient landmarks are more strongly encoded and easier to recall than those associated with non-salient landmarks. In the presence of multiple salient landmarks, multiple intrinsic frames of reference are formed and spatial relations are anchored to each individual frame of reference. Multiple such representations may co-exist and interactively determine a person's spatial performance.

## Introduction

While it is generally accepted that any physical space has to be somehow digested and encoded in a psychological space in order to be cognitively useful, a large body of evidence has convincingly shown that a psychological space is not an exact copy of the corresponding physical space. On one hand, a physical space can be defined as perfectly three-dimensional, absolute, unified, continuous, and Euclidean. On the other hand, it is well documented that the psychological space is often segmented, relative, partial, distorted, and non-Euclidean [1]–[5].

The discrepancies between the physical space and the psychological space manifest themselves in the judgment of geographical information such as locations and bearings [2], [6]–[13]. Stevens and Coupe [12] first proposed that the representations of spatial relations are hierarchically organized. Hirtle and Jonides [6] suggested that people misjudge geographical locations with the biases or distortions appearing to be based on subjective spatial categories. When asked to point out landmarks in a map, participants in Hirtle and Jonides' experiments overestimated distances for between-cluster pairs and underestimated them for within-cluster pairs, suggesting that they formed subjective spatial categories even without seeing well-defined boundaries in the space. The category adjustment model [14]–[15] suggests that people represent spatial locations at more than one level of spatial resolution. Huttenlocher, Hedges, and Duncan [14] showed participants a circle with a dot in it then asked the participants to reproduce the dot position based on memory. They found that the reproduced positions systematically deviated from their original positions. Specifically, if the circle was divided into horizontal-vertical and radial slices, the reproduced dots were often displaced toward the center of the slices in which they fall. This result suggests that a psychological space might be hierarchically represented and there is a central tendency at each hierarchical level (e.g., the slice) which all other spatial information is anchored upon. When memories at the finer level are inexact, categorical information is more heavily weighted and biases can arise.

The hierarchical organization of psychological space is also documented in other tasks of geographical judgment [for a review, see 10]. The view of perceptual heuristics [11], [13] posits that the biases or distortions occur due to heuristics derived from principles of perceptual organization. For example, people tend to misjudge South America to be far more west than it actually is because they “align” North America and South America into a simple unit along a longitudinal axis. In contrast, the view of categorical organization [7]–[9] proposes that people's subjective representation of geographic locations is principally categorical rather than due to perceptual (or figurative) processing. For example, people tend to group North America into four regions (Canada, the northern U.S., the southern U.S., and Mexico). Their estimates showed definite jumps when region boundaries were crossed with little differentiation for estimates within regions. Using hypothetical maps, the experiments by Newcombe and Chiang [10] showed that the learning of geographical information may be more based on perceptual heuristics than on categorical organization. However, they also suggest that it is possible that both perceptual heuristics and categorical organization play roles in learning graphical locations depending on the context in which information is learned. In lack of specific knowledge of categories gained from personal experiences such as from film, TV show and actual travel, geographic representations may be based more on the use of perceptual heuristics than on categorical organization.

In the present study we examined the hierarchical organization of psychological space by people's usage of multiple intrinsic frames of reference. The concept of frame of reference has played an essential role in investigating the psychological representation of physical space and it has been well accepted that different frames of references are utilized in spatial representation, such as egocentric, intrinsic and allocentric systems [16]–[26]. Different from the egocentric system (self-centered) or the allocentric system (referenced to an absolute frame), the intrinsic reference system anchors on the particular objects in the environment. This feature makes the intrinsic reference system particularly relevant in the task of memorizing geographic information from maps (real or hypothetical) and the spatial relations in a layout of objects. More recently, the importance of spatial representations in intrinsic reference systems has gained attention in human spatial behavior [22], [27]–[29]. For instance, Mou and his colleagues demonstrated that inter-object spatial relations were specified with respect to an intrinsic reference direction in the scene. H. Wang et al. [29] show that the updating of spatial relations in intrinsic reference system is affected by the salience level of the object that provides the anchor to the reference system. In the present study, we hypothesize that in memorizing the spatial relations, multiple distinctive intrinsic frames of reference are established based on multiple salient landmarks. Then, the psychological representation of the physical space is distorted and segmented hierarchically due to the co-existence and the interaction between individual reference systems.

Our hypotheses are derived from an integrated theory of human spatial representations called FORMS, “Frame of Reference based Maps of Salience” [29]–[32]. Specifically, if we present to subjects an environment (such as a hypothetical map), in which some objects are made salient, either through top-down influences (e.g., emphasizing their importance by instructions) or bottom-up distinction (e.g., perceptually standing out from other objects), and ask subjects to remember the layout of the environment, then we would expect the encoding of the environment would be anchored around these salient objects. That is, multiple intrinsic frames of reference can be formed in which the origin and axes of the reference systems are anchored on the salient objects. Within individual reference systems, distortions from the physical space arise since the relative locations of these objects are not treated equally in the psychological space. For example, objects that are directly paired with the origin or the axes of the reference system are more strongly associated with each other. Furthermore, segmentation would occur across different reference systems. An object pair belonging to the same reference system would be more strongly associated with each other than an object pair that comes from different reference systems.

Different from previous studies that used accuracy as criterion of biases (such as judgment of distance and bearings), we used reaction times in recalling relative locations as a measure of the distortion and segmentation in the psychological representations of the physical space. Specifically, the experiments used strictly controlled object arrays to examine the roles of salient landmarks and figurative configurations of the physical map (such as the center point). As a result of the formation of distinctive intrinsic reference systems, if we measure the time it takes to retrieve the spatial relations from memory the reaction times should show a symmetrical pattern surrounding the anchoring object (hence referred to as “landmark”). In other words, reaction time is not always proportional to the corresponding physical distance. Rather, it is determined by the “psychological distance” defined within individual reference systems. The retrieval of the spatial relation between object pairs that directly involve the landmarks or belong to the same reference system would be faster than those indirectly involve the landmarks or belonging to different reference systems.

## Results

### Experiment 1

**Figure 1:**
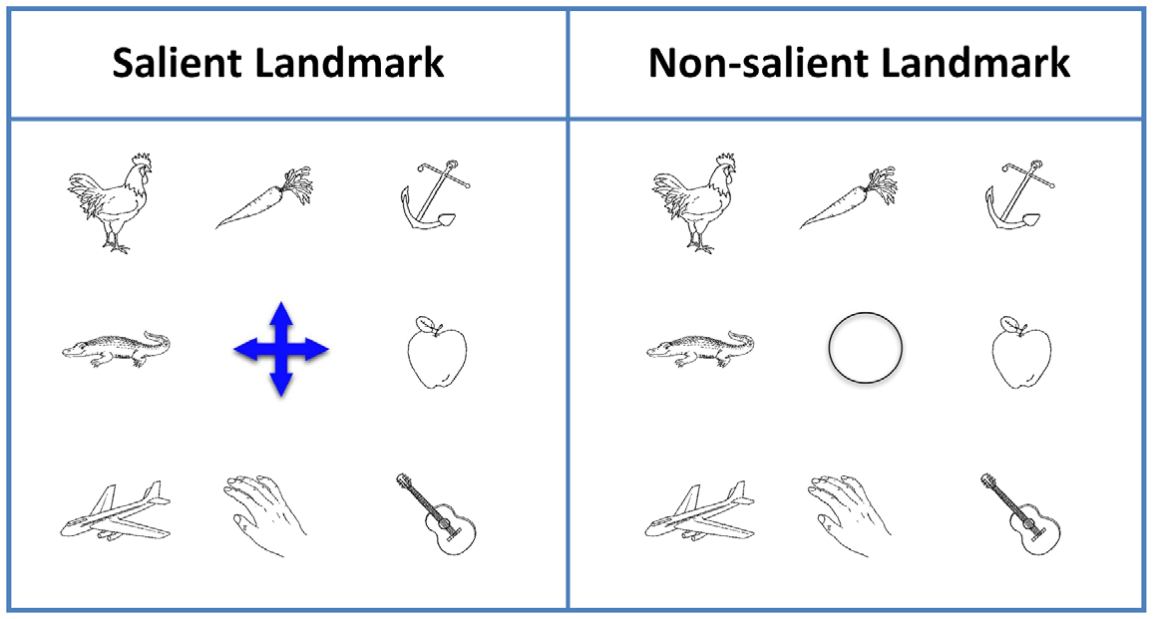
The object layout in Experiment 1. The cross in the middle of the object array in the salient condition was made perceptually distinctive with blue coloring while all other objects were in black-and-white.

Experiment 1 measured the reaction time pattern in a simple object layout and the salience effect of the anchoring object (landmark) in the intrinsic frame of reference (Figure 1).

Among the 28 total possible object pairs in each array, we classified them into 7 groups based on two variables (see Table 1). One was whether the pair involved the landmark, which could be a landmark-object relation (e.g., the chicken and the blue landmark in Figure 1) or a landmark-linked relation (e.g., the chicken and the guitar in Figure 1). The other one was the distance, which could be 1, 1.414, 2, and 2.818 (arbitrary unit, 1 unit is defined as the shortest distance between two adjacent objects).

**Table 1:** The classification of object pairs in Experiment 1.

Group	# of pairs (28 total)	Landmark-linked	Landmark-object	Distance	Example
1	4	no	yes	1	landmark-carrot
2	8	no	no	1	chicken-carrot
3	4	no	yes	1.414	landmark-chicken
4	4	no	no	1.414	crocodile-carrot
5	2	yes	no	2	carrot-hand
6	4	no	no	2	chicken-anchor
7	2	yes	no	2.818	chicken-guitar

**Figure 2:**
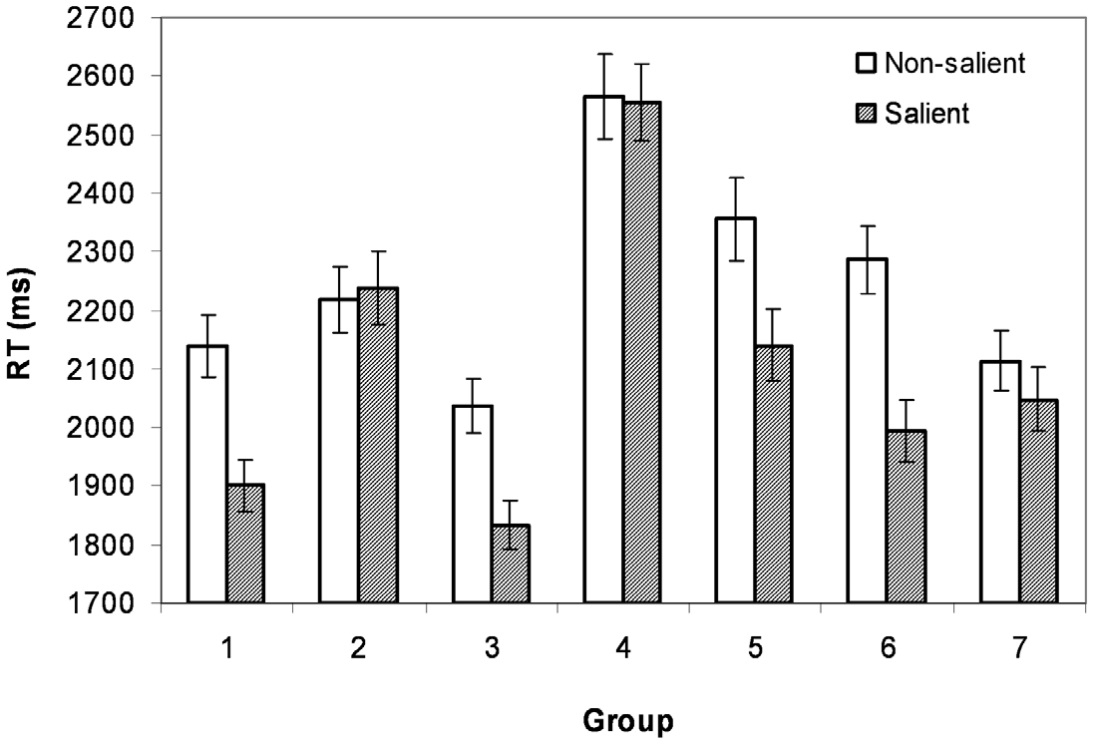
Reaction time in Experiment 1 based on salience and group variables. The error bars are standard errors.

Among twenty subjects the average accuracy was 96.5% with a standard deviation of 4.65%. The subjects' reaction time as a function of the salience condition and group number is shown in Figure 2. The overall statistical analyses showed that both the salience effect (mean difference  = 143.84 ms, F(1, 19)  = 16.03, p<.01, estimated effect size  = .458) and the object grouping effect (F(6, 114)  = 20.36, p<.01, estimated effect size  = .517) were significant, as well as their interaction (F (6, 114)  = 4.72, p<.01).

**Figure 3:**
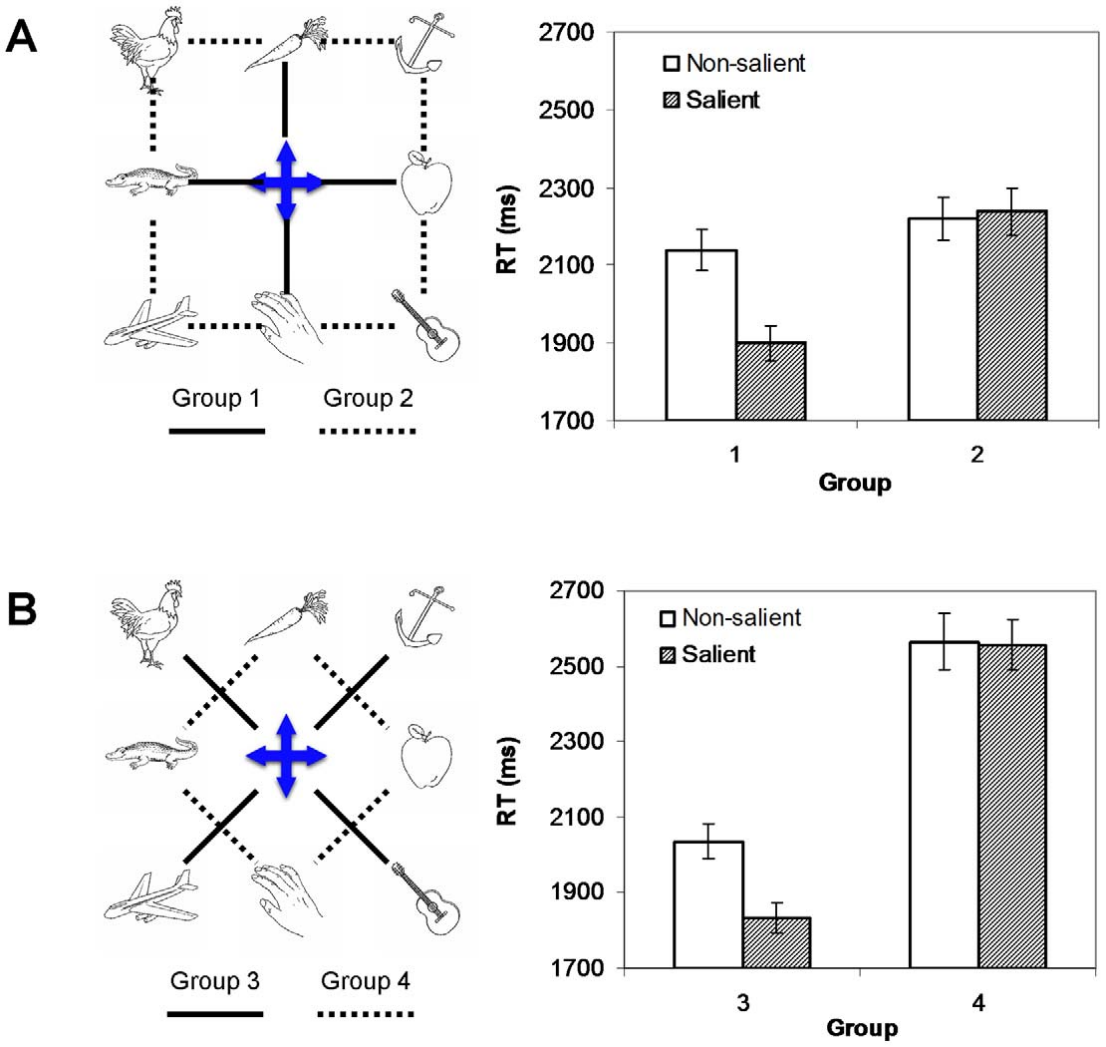
Reaction time comparison between groups. A. Comparison between groups 1 (solid lines) and 2 (dotted lines). B. Comparison between groups 3 (solid lines) and 4 (dotted lines). The error bars are standard errors.

Since our main interest was to examine the role of landmarks rather than the distance, we focus on the comparisons of two pairs of groups with comparable distances. Figure 3A shows the comparison between groups 1 and 2, and Figure 3B shows the comparison between groups 3 and 4. Two major observations can be made from Figures 3A and 3B. First, reflected in reaction times, distortions in the psychological space occurred before introducing the salient landmark. This effect is more apparent in Figure 3B. Comparing groups 3 and 4 on the non-salient condition only, whereas the distance between the object pairs was the same, RT was significantly faster in group 3 in which the object pair contained the central object (mean difference  = 528.8 ms, t(19)  = 5.78, p<.001). Second, introducing a salient landmark not only significantly reduced the overall reaction time but also amplified the distortion. This effect can be observed in Figure 2, which shows the significant main effect of salience and the significant interaction between the salience effect and the group effect. It indicates that the introduction of a salient landmark has significantly changed the psychological representation of the physical map.

Further analyses including all groups showed that only the groups 1, 3, 5, and 6 showed significant salience effect (group 3 has the smallest mean difference of 202.5 ms with a standard error of 54.72) and that the groups 2, 4, and 7 did not. This was what we predicted given that those pairs in those significant groups all involved the landmark, indicating that the spatial representations were organized around the salient landmarks in a hierarchical fashion. This result was especially interesting given that we did not emphasize to the subjects the importance of the central landmark in the encoding and subjects were free to choose their own encoding strategy, an indication that in our experiment, hierarchical organization is induced by perceptual properties.

Together, Experiment 1 clearly showed a distortion pattern in which the associations involving the center object were stronger than other associations, as if the psychological space had been “compressed” towards the central. Such distortion became significantly greater when the saliency level of the central object was increased.

### Experiment 2

Experiment 2 extended Experiment 1 in a more complex environment for two purposes. First, besides the distortions in the psychological space within a single frame of reference, we also hypothesize that distortions will be segmented and arise across multiple frames of reference. Thus, it remains to be tested whether multiple frames of reference will be constructed given multiple salient landmarks and how distortions would occur correspondingly. Second, the salient landmark was positioned at the center of the object array in Experiment 1 and it showed that the central location alone without a salient landmark can distort the reaction time pattern. We need to compare the relative roles of the salient landmarks and the natural figurative configuration of the physical map during the construction of the frames of reference.

**Figure 4:**
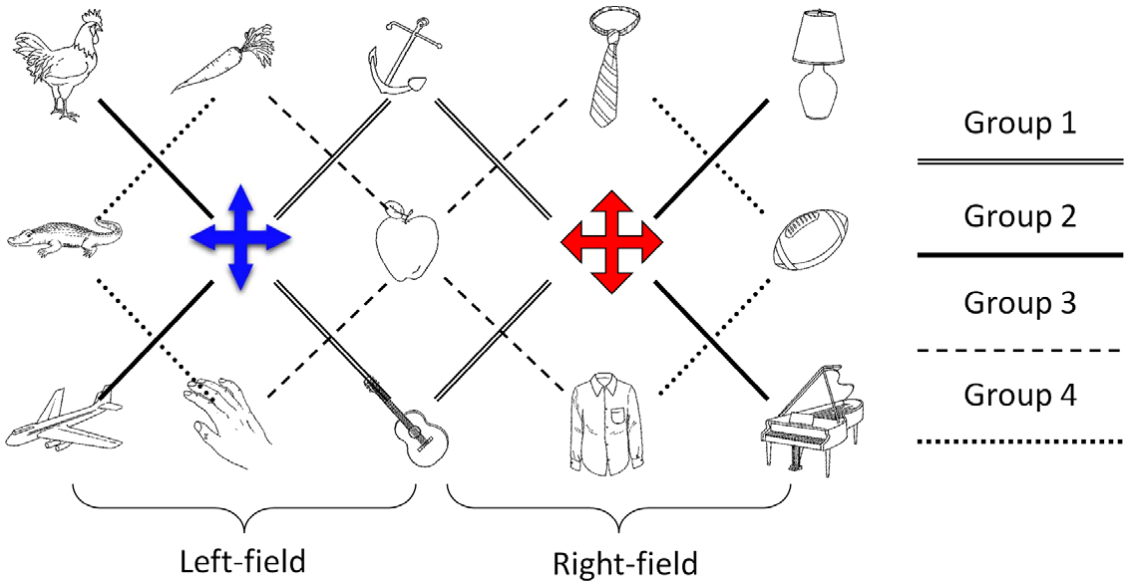
The object layout and the four focus groups in Experiment 2. The left cross is in blue and the right cross is in red. All other objects are in black-and-white.

**Figure 5:**
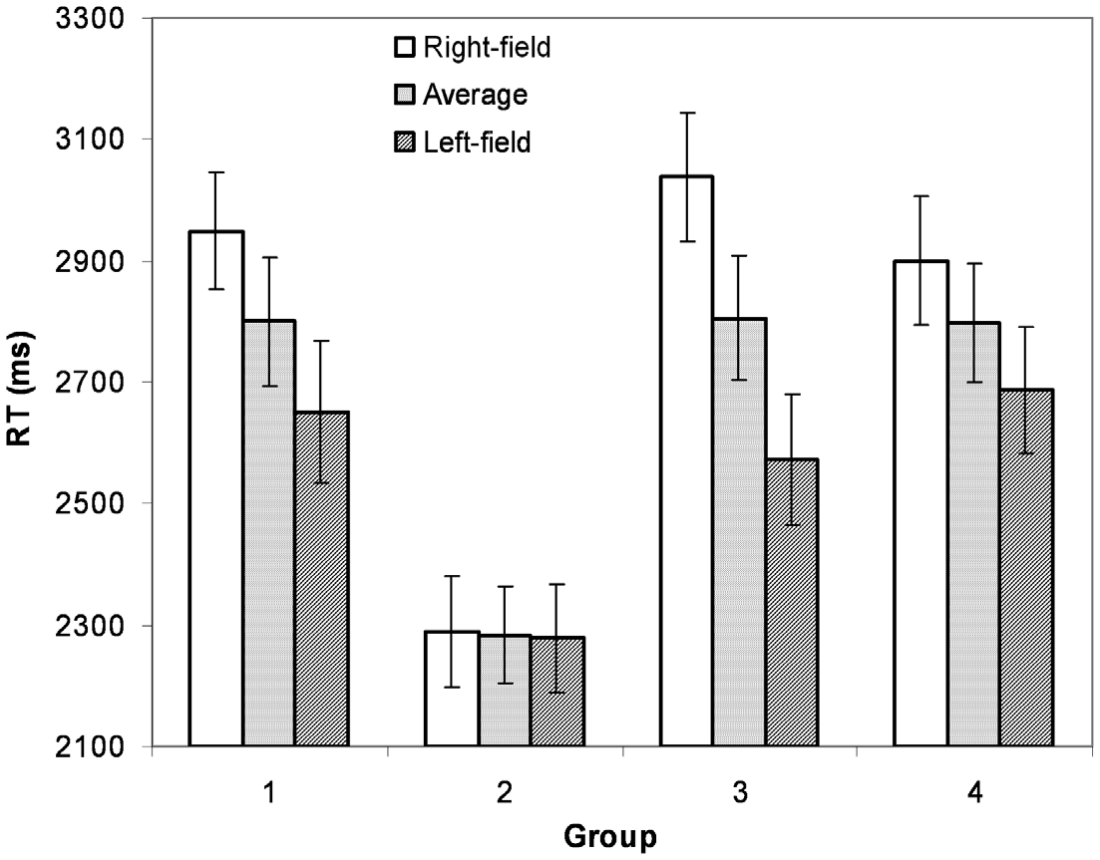
Reaction times in Experiment 2 based on field and group variables. The error bars are standard errors.

Among eleven subjects the average accuracy was 96.8% with a standard deviation of 2.44%. Since the size of the object array has almost been doubled, the possible combinations of object pairs in Experiment 2 were much more complicated than in Experiment 1. To simplify, we focused on 4 groups of object pairs with the same between-pair distances. Figure 4 shows these focus groups. Group 1 (double lines) represents objects paired with both of two salient landmarks. Group 2 (solid lines) represents objects paired with only one of the salient landmarks. Group 3 (dashed lines) represents objects linked to the central object but not the salient landmarks. Group 4 (dotted lines) represents connections between objects without involving either landmarks or the central object. Furthermore, the entire array is divided into the left-field and the right-field from the middle and both fields include the central column of objects. Based on the 4 focus groups illustrated in Figure 4, the subjects' reaction times are presented in Figure 5, in which mean reaction times for the left-field, the right-field, and the average of two fields are presented separately.

We examined the group effect, the field effect (left vs. right), and the interaction by a two-way repeated-measures ANOVA. Both of the group effect and the field effect were statistically significant (F(1,11)  = 12.275, p<.01, F(3,33)  = 5.336, p<.01, respectively), but the interaction between groups and fields was not (F(3,33)  = .719, p = .548). This result suggests three overall patterns. First, measured by the reaction time, the psychological space has been distorted significantly depending on the relative locations of the object pairs, specifically, whether the object pairs contain the landmarks or the central point. Second, object locations in the left field were better memorized than in the right field. This probably was due to the left-to-right reading habit and the object pairs viewed first would be more strongly encoded. Third, the non-significant interaction between groups and fields showed that the distortion induced by groups was very similar in two half fields, indicating a hierarchical representation system with two sub-systems splitting the entire map into the left and right halves.

To further investigate the roles of the landmarks and the central point, we conducted 4 comparisons between 4 pairs of groups, groups (2, 4), groups (1, 2), groups (2, 3) and groups (3, 4), over the averaged reaction times of two half fields. First of all, the reaction time from group 2 was significantly faster than from group 4 (mean difference  = 514.8 ms, t(10)  = 3.64, p<.005). This basically replicates the findings from Experiment 1 that within a single frame of reference, an object was more strongly associated with the salient landmark than with other objects. Second, the reaction time from group 1 was significantly slower than from group 2 (mean difference  = 519.3 ms, t(10)  = 4.39, p<.001). This indicated that when an object can be referenced to two landmarks instead of one, the reaction was significantly slowed down. The reason may be that referencing an object to multiple frames of reference causes multiple representations competing with each other, and selecting from or switching between multiple systems takes time. Third, the reaction time from group 2 was significantly faster than from group 3 (mean difference  = 521.4 ms, t(10)  = 3.96, p<.003). This suggests that the association of an object with the landmark was significantly stronger than the association of an object with the central object. The weaker role of the central object was further confirmed by the fourth comparison between groups (3, 4), which was not statistically significant (mean difference  = 6.6 ms, t(10)  = .06, p = .95), indicating that in the presence of the salient landmarks, the central object received little special attention compared with other objects.

**Figure 6:**
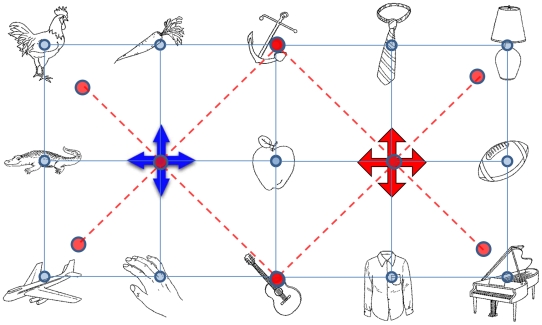
Segmentation and distortion of psychological space described by reaction times. Reaction times in recalling spatial relations of object pairs are not proportional to the distances in the physical space. The relative lengths of the dotted lines represent the comparison of reaction times when the object is associated with a single landmark (group 2 in Figure 4) and when the object can be associated with two landmarks (group 1 in Figure 4), with the latter as the base length.

In summary, Experiment 2 confirmed our predictions of a hierarchical and segmented organization with distinctive frames of reference based on the salient landmarks (reference points). Distortions arise in the psychological space in the sense that reaction times in recalling the relative locations of object pairs were not proportional to the distances in physical space (Figure 6). The symmetrical distortion patterns in the left and right half fields indicated the existence of two individual sub-level reference systems and each was formed around its own salient landmark. Within each sub-system, distortion occurred surrounding the landmark respectively. The two sub-systems competed with each other when an object could be referenced to both of them.

## Discussion

In this paper, we presented two experiments to test the predictions of salience-based hierarchical spatial representations by multiple intrinsic frames of reference. Our hypotheses were derived from an integrated theory of human spatial representation called FORMS. FORMS essentially states that a psychological space consists of multiple representations, each with a distinctive frame of reference and each being only a subset of all possible spatial information. The two experiments reported here used reaction times instead of the accuracy of the recalled relative locations as a measure of the distortions in the psychological representations of the physical space. The experiments used strictly controlled object arrays to examine the roles of salient landmarks and figurative configurations of the physical map, and they confirmed the claims of FORMS. The results suggest that at least two factors are responsible for inducing distinctive frames of reference in our experiments. One is the overall figurative layout of the entire map and the other is based on salient landmarks. These two factors will reinforce each other if they work together. In our experiment 1, the non-salience condition alone produced significant distortions surrounding the central object. When introducing the salient landmark in the salience condition, such distortion was even greater.

The key concepts in our account are the salience level of the objects that serve as the bases (origin and axis) of the intrinsic frame of reference and the existence of multiple reference systems. These two concepts are consistent with the category adjustment model [14]–[15] and provide connections between the mechanisms of perceptual heuristics [11], [13] and categorical organization [7]–[9] (also see [10]). That is, the formation of multiple reference systems can be determined by both perceptual heuristics (bottom-up) or categorical knowledge (top-down). Once the intrinsic frames of reference are established, the regionalization/categorization are a result of organization according to different reference systems. For example, it appears that the Experiment 4 in Newcombe & Chiang [10] did not induce distinctive salience-based categories (sub-level reference systems). That is, the efforts to increase conceptual processing and categorical organization by coding the regions into different colors and different climate groups were not effective in producing categorical organization. The reason may be that salient anchors (landmarks) were not provided within the regions so that distinctive frames of reference are not induced corresponding to the individual regions. Indeed, this speculation is confirmed by the Experiment 5 in Newcombe and Chiang [10]. It showed that the provision of two correctly placed locations allowed people to place other cities with excellent accuracy. This is consistent with the findings in our Experiment 2 in that the salient landmarks were the driving force in constructing distinctive frames of references.

In essence, our hypotheses are founded on the construction of multiple distinctive salience-based reference systems to account for the distortions or biases in the psychological space. Particularly, the salience effect attributes to the distortions not only within but also between reference systems. Saliency can be achieved either through top-down instructions or existing knowledge or bottom-up perceptual distinction. Then, categorical organizations are in effect representations in a hierarchy of multiple reference systems around salient reference points. Within the same reference system, distortions can occur because of the role of the salient reference point. Furthermore, errors and interferences can arise when people switch between different reference systems.

## Methods

### Experiment 1

#### Participants

Twenty college students and graduate students in the Houston medical center area participated in Experiment 1 for pay. The experiments were conducted in accord with APA standards for ethical treatment of subjects and with the approval of IRB at University of Texas Health Center at Houston. Informed consent was obtained from all participants in written form.

#### Procedure

Two conditions were compared by a repeated-measure design, 4 blocks of trials for each condition. In each block, subjects were first presented with an evenly spaced 3×3 array of objects on a computer screen. In the salient condition, the central object in the array was perceptually distinct from all others (Figure 1, left panel), and in the non-salient condition, a non-distinctive object was used instead (Figure 1, right panel). The order of two conditions was counter-balanced between subjects. Subjects were asked to remember the spatial layout of these objects (self-paced). After the study phase, we tested subjects' memory by presenting pairs of objects on the center of the screen and asking subjects to decide if the relative location between the pair was the same as in the study phase. The test in each block consisted of 28 object pairs that were aligned horizontally, vertically, or diagonally in the original layout. Each object pair was tested twice, once positively and once negatively, in which the relative location of the object pair in the test phase was either the same as or different from that in the study phase. In total, each subject completed 448 trials (28×2×8 blocks). The trials in the test phase were randomized. The reaction time data were recorded.

### Experiment 2

#### Participants

Eleven college students and graduate students in the Houston medical center area who were not included in Experiment 1 participated in Experiment 2. Participants were paid for participation. The experiments were conducted in accord with APA standards for ethical treatment of subjects and with the approval of IRB at University of Texas Health Center at Houston. Informed consent was obtained from all participants in written form.

#### Procedure

Figure 4 illustrates an example layout of the experiment setup. Compared to Experiment 1, the object array in Experiment 2 was increased in size from 3×3 to 3×5. All objects were still evenly spaced. Two salient landmarks were positioned at the coordinates (2,2) and (2,4), separate from the center location of the entire layout at (2,3). Since we had already established the salience effect in Experiment 1, Experiment 2 did not vary the salience level of the landmarks. As in Experiment 1, subjects were asked to remember the spatial layout of the objects (self-paced). After the encoding, we tested subjects' memory by presenting pairs of objects on the screen and asking subjects to decide if the pair was in its originally relative relations. Each subject was tested on 2 object arrays. In each array, 58 object pairs were tested (horizontal, vertical and diagonal pairs only) and each pair was tested once positively and once negatively. In total, each subject completed 232 trials (58×2×2 blocks). The reaction time data were recorded.
